# Characterization of *Argopecten purpuratus* Shells as Marine-Derived Bioceramics: Microstructural and Biological Insights for Tissue Engineering Applications

**DOI:** 10.3390/jfb17040164

**Published:** 2026-04-01

**Authors:** Carola Millán, Isabel Benjumeda-Wijnhoven, José I. Contreras Raggio, Astrid Muñoz, Ignacia Muñoz-Brautigam, María F. Álamos, Marco A. Lardies, Juan F. Santibañez, Nelson A. Lagos, Juan F. Vivanco

**Affiliations:** 1 Facultad de Artes Liberales, Universidad Adolfo Ibáñez, Viña del Mar 2520000, Chile; carola.millan@uai.cl (C.M.); isabel.benjumeda@uai.cl (I.B.-W.); marco.lardies@uai.cl (M.A.L.); 2 Facultad de Ingeniería y Ciencias, Universidad Adolfo Ibáñez, Viña del Mar 2520000, Chile; 3 Institute for Medical Research, National Institute of the Republic of Serbia, University of Belgrade, 11221 Belgrade, Serbia; jfsantibanez@imi.bg.ac.rs; 4 Centro de Investigación e Innovación para el Cambio Climático (CiiCC), Facultad de Ciencias, Universidad Santo Tomás, Santiago 2270000, Chile; nlagoss@santotomas.cl

**Keywords:** natural biomaterials, microstructural characterization, marine-derived calcium carbonate, cell–material interactions, stem cell adhesion and proliferation, bone tissue engineering

## Abstract

This study presents a comprehensive characterization of *Argopecten purpuratus* (*AP*) shells—a marine-derived natural bioceramic composed predominantly of calcium carbonate (CaCO_3_)—to evaluate their potential as biomaterials for regenerative medicine. Structural and compositional analyses were performed using micro-computed tomography (MicroCT), scanning electron microscopy (SEM), Fourier-transform infrared spectroscopy (FTIR), thermogravimetric analysis (TGA), and X-ray diffraction (XRD). These techniques confirmed a high CaCO_3_ content (>96 wt%) and revealed distinct microstructural features: the outer surface showed irregular grooves and rough textures, while the inner surface exhibited smoother, foliated morphologies with mixed calcite and aragonite phases. To assess biocompatibility, human gingival mesenchymal stem cells (hGMSCs) were cultured on both shell surfaces. Viability and adhesion were evaluated via MTS assays and fluorescence microscopy at time points ranging from 30 min to four weeks. Both surfaces supported robust early metabolic activity and long-term proliferation, with cells covering the entire surface area after four weeks. Morphometric analysis indicated time-dependent changes in cell shape, transitioning from rounded to elongated morphologies, with minor differences linked to surface topography. The integration of structural, compositional, and biological data demonstrates that AP shells provide a cytocompatible and sustainable natural material platform capable of supporting cell adhesion and proliferation. Their inherent micro- and nanoscale surface features may facilitate protein adsorption and cell–material interactions. These findings highlight the importance of correlating microstructural material properties with cellular responses and support the future exploration of marine-derived bioceramics for regenerative medicine applications.

## 1. Introduction

The development of sustainable, high-performance biomaterials has driven significant interest in naturally derived structures that exhibit complex hierarchical organization and favorable physicochemical properties. Mollusk shells, primarily composed of calcium carbonate (CaCO_3_) minerals in aragonite and calcite polymorphs embedded within an organic biopolymer matrix, demonstrate a multilayered architecture that imparts exceptional mechanical strength, resistance to crack propagation, and osteoinductive potential [1,2]. This unique combination of structural and chemical features has positioned mollusk shells as promising candidates for applications within materials science and biomedical engineering, particularly in the design of scaffolds for tissue regeneration [3,4,5].

Marine-derived biomaterials such as shells from *Argopecten purpuratus* (scallop) provide an abundant and underutilized source of CaCO_3_-based biominerals with structural and compositional characteristics closely resembling natural bone tissue [3,4,6]. The shell’s hierarchical organization, which varies between the outer and inner surfaces, presents diverse morphologies and textures that are critical for their functional performance. These surfaces exhibit differences in roughness, porosity, and microstructure, which can influence cellular behavior when used as substrates for tissue engineering applications. Such natural complexity is often difficult to replicate in synthetic materials, highlighting the importance of detailed characterization to fully understand their potential utility [7].

Calcium phosphate (CaP)-based bioceramics, especially hydroxyapatite, have traditionally dominated the field of bone tissue engineering due to their bioactivity and osteoconductivity [8,9]. However, their relatively high production costs, brittleness, and mechanical mismatch compared to native bone tissue have limited their broader application [10]. Consequently, there is a growing interest in alternative biomaterials that combine comparable chemical and mechanical properties with enhanced surface topographies and improved biological integration. Hence, marine-derived CaCO_3_ materials present a cost-effective and sustainable alternative that warrants extensive exploration.

The surface morphology and microstructural features of biomaterials are well-recognized determinants of protein adsorption, cell adhesion, proliferation, and differentiation [11,12]. Cellular adhesion is a complex and dynamic process mediated by transmembrane and cytoplasmic proteins that interact with specific ligands on the material surface, enabling cells to establish anchorage and initiate signaling pathways crucial for tissue regeneration [13,14]. Numerous studies have demonstrated that surface roughness and topography at both the micro- and nanoscale critically affect cell behavior, influencing the extent and quality of cellular attachment and subsequent proliferation [15,16]. Notably, biomimetic calcite topographies have been proposed as promising templates for tissue engineering, offering the potential to direct cellular responses through their inherent structural cues [17].

Given this context, comprehensive characterization of the morphological and microstructural properties of natural biomaterials is essential to correlate surface features with biological outcomes. Techniques such as scanning electron microscopy (SEM) and micro-computed tomography (microCT) enable detailed visualization and quantification of surface textures, porosity, and internal architecture, providing insights that inform material selection and scaffold design [6,7]. Despite growing interest, few studies have simultaneously examined the morphological features of scallop shells and their interactions with relevant stem cell populations, such as human gingival mesenchymal stem cells (hGMSCs), which possess multipotent differentiation capabilities suitable for regenerative medicine [5,7].

This study aims to bridge this gap by conducting a thorough morphological and microstructural assessment of *Argopecten purpuratus* shells, focusing on the distinct inner and outer surfaces, and evaluating their biocompatibility with human gingival mesenchymal stem cells (hGMSCs). By combining advanced imaging techniques with cellular assays, we seek to elucidate how shell surface features influence initial cell adhesion and viability, as well as the morphological evolution and proliferation of hGMSCs over extended culture periods. Understanding these interactions will contribute to the rational design of marine-derived biomaterials with optimized structural and biological properties, supporting their application in bone tissue engineering and regenerative medicine.

## 2. Materials and Methods

### 2.1. Processing of Argopecten purpuratus Shell Fragments

Chilean scallop shells (*Argopecten purpuratus*) were obtained from juvenile *Argopecten purpuratus* scallops with mean shell length of 41 ± 1.3 mm (mean ± SD), collected from Invertec Ostimar Co. scallop farm located in Tongoy Bay (30° S), central Chile. Shells were treated with ammonia 50% *v*/*v*, then washed with distilled water, cleaned with a metal sponge, and then boiled in distilled water for 20 min to remove any adhered organic residues. Afterwards, shells were air-dried and cut into fragments using a bone saw to an average area size of 12 mm^2^. Samples were sterilized with 95% alcohol, dried for 60 min at 90 °C, and exposed to 30 min UV radiation. Micro computed tomography (Micro-CT) was conducted to characterize the fragment micro architecture. Images were obtained in an EasyTom (Rx Solutions, Chavanod, France) at 80 kV and 200 µA, and analyzed with Avizo Fire (Thermo Fisher Scientific, Waltham, MA, USA).

The boiling and ammonia treatment steps were applied to remove residual organic matter and biological contaminants naturally present on the shell surface. These procedures are commonly used for shell cleaning and are intended to preserve the mineral structure of calcium carbonate. To verify that the processing did not induce significant chemical or structural alterations, the treated shells were characterized using thermogravimetric analysis (TGA), Fourier transform infrared spectroscopy (FTIR), X-ray diffraction (XRD), and scanning electron microscopy (SEM).

### 2.2. Thermogravimetric Analysis (TGA)

Thermogravimetric analysis (TGA) was conducted to evaluate the thermal stability and compositional profile of the *Argopecten purpuratus* shell material. The analysis was performed using a TG 209 F1 thermobalance (Netzsch, Selb, Germany), operating under a controlled atmosphere. Samples were heated from room temperature up to 900 °C at a constant rate of 10 °C/min. The mass loss was recorded as a function of temperature to identify decomposition stages and quantify the organic and inorganic constituents of the shells. This analysis provided insight into the presence of organic matrix components, moisture content, and the thermal behavior of the calcium carbonate phases.

### 2.3. Fourier Transform Infrared Spectroscopy (FTIR)

Fourier transform infrared spectroscopy (FTIR) was employed to analyze the chemical composition and functional groups present on the surface of the *Argopecten purpuratus* shell fragments. This technique provides qualitative insight into the molecular bonds and mineral phases, particularly the carbonate structure characteristic of calcium carbonate-based biominerals.

Spectra were recorded using a Cary 640 FTIR spectrometer (Agilent Technologies, Santa Clara, CA, USA) equipped with a DTGS detector. Finely ground shell samples were mixed with spectroscopic-grade KBr and pressed into pellets. The spectra were acquired in the range of 4000–500 cm^−1^ with a resolution of 4 cm^−1^. Data acquisition and processing were performed using Agilent Resolutions Pro software (version 5.2.0). The resulting spectra were analyzed to identify characteristic absorption bands associated with calcite or aragonite polymorphs, organic components, and potential impurities within the bioceramic matrix.

### 2.4. X-Ray Diffraction (XRD)

X-ray diffraction (XRD) analysis was carried out to determine the crystalline phases and structural composition of both the inner and outer surfaces of the *Argopecten purpuratus* shell samples. This technique was used to identify the mineralogical polymorphs of calcium carbonate, primarily aragonite and calcite, which are characteristic of marine bioceramics.

XRD patterns were obtained using a Panalytical X’Pert Pro diffractometer (Malvern Panalytical, Almelo, The Netherlands) equipped with a CuKα radiation source (λ = 1.5406 Å) and a graphite monochromator. Measurements were performed in reflection mode over a 2θ range of 15–60°, with a step size and counting time optimized for reliable phase identification. The resulting diffractograms were analyzed to determine the relative abundance and crystallographic orientation of the observed phases, allowing comparison between the microstructural organization of the inner and outer shell surfaces.

### 2.5. Human Gingival Mesenchymal Stem Cells (hGMSCs) Cell Culture

Primary human gingival mesenchymal stem cells (hGMSCs) were obtained from healthy adult donors following informed consent and ethical approval by the Institutional Review Board of Universidad Adolfo Ibáñez (IRB-UAI), under the Bioethical Research Committee (Approval No. 54/2019), as described in ref. [18]. All procedures involving human-derived cells were conducted in compliance with the Manual of Biosafety Standards and Associated Risks and were approved by IRB-UAI to ensure safe handling and ethical standards.

Cells were propagated between passages 3 and 8 to maintain multipotency and minimize phenotypic drift. hGMSCs were cultured in Dulbecco’s Modified Eagle Medium (DMEM, Gibco, Waltham, MA, USA) supplemented with 10% fetal bovine serum (FBS, Gibco, Waltham, MA, USA) and 1% Penicillin–Streptomycin (Genesee Scientific, El Cajon, CA, USA). Cultures were maintained at 37 °C in a humidified atmosphere containing 5% CO_2_ and 95% air. Cells were expanded in T75 culture flasks and used for experiments once they reached approximately 80% confluence.

For subculturing, cells were detached using 0.25% trypsin-EDTA, centrifuged, and resuspended in fresh medium. Cell concentration and viability were assessed using a LUNA-II™ automatic cell counter (Logos Biosystems, Anyang-si, Korea). Additionally, cell viability was cross-verified via the 0.4% trypan blue exclusion method to ensure consistent quality and reproducibility prior to seeding onto shell substrates.

### 2.6. Culture Plate Preparation and Seashell Fragment Treatment

To promote selective cell attachment to the *Argopecten purpuratus* seashell fragments rather than to the underlying plastic surface of the culture plates, a protocol involving agarose coating was implemented. A 2% (*w*/*v*) agarose solution (Sigma, St. Louis, MO, USA) was prepared in phosphate-buffered saline (PBS), following the procedure outlined in [19].

Within a sterile cell culture hood, 50 µL of the agarose solution was carefully dispensed into each well of 96-well plates to form a thin, non-adherent hydrogel layer. The agarose-coated plates were then sterilized by exposure to ultraviolet (UV) radiation for 30 min to ensure aseptic conditions. Subsequently, 50 µL of Dulbecco’s Modified Eagle Medium (DMEM) was added to each well to precondition the surface prior to cell seeding.

Separately, seashell fragments were sterilized and incubated in DMEM for one hour to equilibrate the material and remove any residual contaminants. Following this conditioning step, individual seashell fragments were aseptically transferred into the prepared wells using sterilized forceps, with one fragment placed per well. This preparation ensured that human gingival mesenchymal stem cells would preferentially adhere to the seashell substrate during subsequent culture experiments, minimizing background cell growth on the plastic surface.

### 2.7. hGMSCs Seeding on Seashell Fragments

Following preparation of the seashell fragments, confluent human gingival mesenchymal stem cells (hGMSCs) were detached from culture flasks using 0.05% trypsin-EDTA (Trypsin 0.05%, 1 mM EDTA in PBS), then centrifuged at 400× *g* for 5 min and resuspended in phosphate-buffered saline (PBS). A defined cell suspension containing 5 × 10^4^ cells in 50 µL of DMEM was carefully seeded directly onto the surface of each seashell fragment.

The seeded fragments were incubated under standard culture conditions (37 °C, 5% CO_2_, 95% humidity) for 30 min to facilitate initial cell attachment. After this adhesion period, the culture volume was gently increased to 150 µL by adding fresh DMEM to provide nutrients and maintain an optimal environment for cell viability and proliferation.

Cell-seeding experiments were conducted at multiple time points, including 30 min, 2 h, 24 h, and 4 weeks, to monitor early attachment, viability, morphological changes, and long-term proliferation on the shell substrates. Negative controls consisted of seashell fragments without cell seeding to assess background signal and material properties, while positive controls involved cells seeded on standard tissue culture plastic wells without agarose coating to evaluate baseline adhesion and growth behavior.

### 2.8. Cellular Viability by MTS Assay

Cell viability was assessed using the MTS assay (Promega, Madison, WI, USA), which measures the metabolic activity of living cells based on their ability to reduce the tetrazolium compound MTS into a soluble formazan product. This colorimetric assay provides an indirect quantification of viable cells adhered to the seashell fragments.

The assay was performed at three time points—30 min, 2 h, and 24 h post cell seeding—to monitor early and intermediate cell viability on the shell substrates. Prior to the assay, culture medium was carefully aspirated from each well, followed by three washes with phosphate-buffered saline (PBS) to remove non-adherent cells and residual media components.

Seashell fragments were then transferred to new 96-well plates pre-coated with agarose to prevent cell attachment to the plastic surface during the assay. Subsequently, an MTS solution was prepared by mixing 120 µL of MTS reagent with 100 µL of DMEM and added to each well containing a seashell fragment.

Following a 2-h incubation period at 37 °C under standard culture conditions, 100 µL of the supernatant containing the formazan product was transferred to a fresh 96-well plate for absorbance measurement. Optical density was recorded at 490 nm using a Synergy HT microplate reader (BioTek, Winooski, VT, USA). The absorbance values correlate directly with the number of metabolically active cells, allowing quantitative comparison of cell viability across time points and between surfaces.

### 2.9. Cell Morphology, Adhesion, and Distribution Analysis

To assess the adhesion and morphological characteristics of human gingival mesenchymal stem cells (hGMSCs) on the surface of *Argopecten purpuratus* shell fragments, a combination of scanning electron microscopy (SEM) and fluorescence imaging techniques was employed.

#### 2.9.1. Scanning Electron Microscopy (SEM)

Cell attachment and surface interaction were first evaluated using SEM at three time points: 30 min, 2 h, and 24 h post-seeding. Shell fragments seeded with hGMSCs were fixed in a buffered solution of 2.5% glutaraldehyde and 0.1 M sodium cacodylate for 2 h at room temperature. Samples were subsequently washed three times in 0.1 M sodium cacodylate buffer for 5 min each and dehydrated through a graded ethanol series (50%, 70%, 95%, and twice in 100%), with each step lasting 5 min.

Following dehydration, the samples were dried using a critical point dryer (Autosamdri-815, Series A), then coated with a thin gold layer using a sputter coater (Desk V, Denton Vacuum, Moorestown, NJ, USA). SEM observations were conducted using a JEOL JSM IT300LV microscope (Akishima, Tokyo, Japan) at various magnifications to visualize the extent of cellular attachment, spreading, and surface integration on both inner and outer shell surfaces.

#### 2.9.2. Fluorescence Microscopy and Morphological Assessment

To further analyze cell morphology and spatial distribution over time, cytoskeletal and nuclear staining was performed on hGMSCs cultured on shell fragments for 30 min, 2 h, 24 h, 48 h, and 4 weeks. Following PBS washes, cells were fixed with 4% paraformaldehyde (PFA) for 20 min at room temperature, then washed three times with PBS containing 0.1% Triton X-100 (PBS-T) for 10 min each.

Actin cytoskeletons were labeled with Phalloidin conjugated to Tetramethylrhodamine B isothiocyanate (TRITC, Sigma Aldrich, St. Louis, MO, USA), prepared at a 1:1000 dilution in 2% bovine serum albumin (BSA) with 0.2% Triton X-100. A volume of 100 µL was added per well, and samples were incubated overnight at 4 °C. The next day, fragments were washed three times with PBS. Nuclei were stained using Hoechst 33342 (1:1000 in PBS, Sigma-Aldrich, St. Louis, MO, USA) for 10 min, followed by additional PBS washes.

Following staining, samples were preserved in 0.4% PFA and stored at 4 °C until imaging. Fluorescence images were captured using an Olympus BX4 epifluorescence microscope or a Nikon C1 Plus confocal microscope (Melville, NY, USA). For confocal analysis, Z-stack images were acquired at 10 µm intervals through the depth of the shell surface. Maximum intensity projections were generated from each stack to evaluate cell coverage, morphology, and distribution across different regions of the biomaterial.

This dual approach—combining SEM and fluorescence microscopy—enabled high-resolution analysis of cell–surface interactions and provided insight into the dynamics of stem cell attachment, spreading, and proliferation on marine bioceramic substrates over time.

#### 2.9.3. Imaging Characterization

Quantitative and qualitative analyses of cell morphology and distribution were performed using fluorescence microscopy and scanning electron microscopy (SEM) image data. These imaging approaches enabled the assessment of hGMSC shape dynamics, cytoskeletal organization, and overall cell–substrate interactions over time.

Fluorescence microscopy images of Hoechst- and Phalloidin-labeled cells were acquired at 30 min, 2 h, and 24 h post-seeding. Hoechst staining (blue) was used to visualize cell nuclei, while Phalloidin-Tetramethylrhodamine (red) labeled filamentous actin (F-actin) in the cytoskeleton. These fluorescent markers enabled detailed evaluation of cell positioning, spreading, and morphological progression on both the inner and outer surfaces of the *Argopecten purpuratus* shells.

Quantitative image analysis was performed using ImageJ software (version 1.52a, National Institutes of Health, Bethesda, MD, USA). Following image preprocessing—including background subtraction, brightness/contrast enhancement, and application of appropriate thresholding—individual cells were segmented and analyzed using particle analysis tools.

Four morphological descriptors were measured to assess cell morphodynamics: (a) *Roundness*, indicating deviation from a perfect circle; (b) *Aspect Ratio *(*AR*), defined as the ratio of the major to minor axis lengths; (c) *Circularity*, reflecting how close the shape is to a perfect circle (with 1.0 being a circle); and (d) *Solidity*, representing the proportion of the convex hull area occupied by the cell.

These parameters provide insight into how cells change shape during attachment and spreading, as described in prior studies [8,20,21].

In parallel, SEM images were qualitatively analyzed to examine ultrastructural details of cell attachment and surface interactions, with particular focus on cell morphology and distribution across different shell topographies. Together, these imaging techniques contributed to a comprehensive evaluation of cell–material interactions at both microstructural and cellular levels.

### 2.10. Statistical Analysis

To evaluate the interaction effect of shell surface (inner–outer) and time (30 min, 2 h, 24 h) upon cell viability based on adhesion to the surfaces (optical density) and changes upon cell morphology, Analysis of Variance (ANOVA) and Tukey’s Honest Significant Difference (HSD) post hoc test were conducted using R (version 4.1.2). The significance level was established at α = 0.05, with normality assessed using the Shapiro–Wilk test and homoscedasticity verified through the Levene Test. In cases where at least one of the assumptions was not met, the Friedman test and Wilcoxon ranks were employed to determine if significant differences existed.

## 3. Results

### 3.1. Argopecten purpuratus (AP) Presents Different Morphological Properties on the Outer and Inner Shell Surfaces

**Figure 1:**
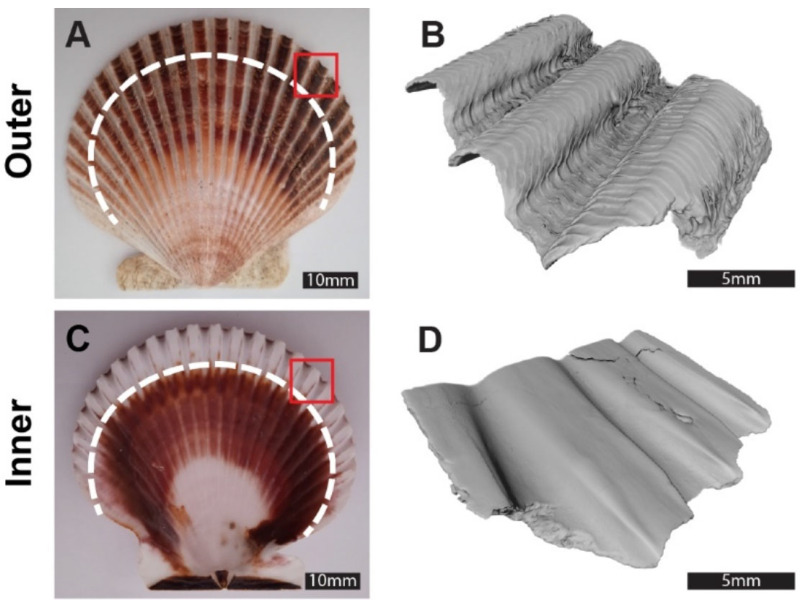
Comparative morphology of the *Argopecten purpuratus* shell’s inner and outer surfaces. Panels (**A**,**C**) show the inner and outer surfaces of the shell, respectively, with the dashed line indicating the outer segment selected for analysis. Panels (**B**,**D**) feature microtomography images of the corresponding segments highlighted in red in (**A**,**C**). The outer shell surface (**B**) displays a rough, uneven texture, whereas the inner surface (**D**) is comparatively smooth and flat. Scales bar are shown in each figure.

The surface characteristics of a substrate establish the initial topological conditions for potential cell adhesion. Samples were collected from the exterior section of the AP shells (Figure 1A,C), with average areas of 13.70 ± 6.31 mm^2^ for the outer surface and 12.08 ± 5.08 mm^2^ for the inner surface. MicroCT scanning revealed distinct morphological features between the outer and inner surfaces as shown in Figure 1B,D. The outer shell face exhibited radial ribs, resulting in a surface with pronounced grooves and scales that create deeper indentations along the elevated areas. This rough topography reflects growth events occurring at the shell’s edge (Figure 1B). In contrast, the inner shell layer displayed a smoother topography, characterized by wave-like patterns with smaller amplitudes (Figure 1D).

**Figure 2:**
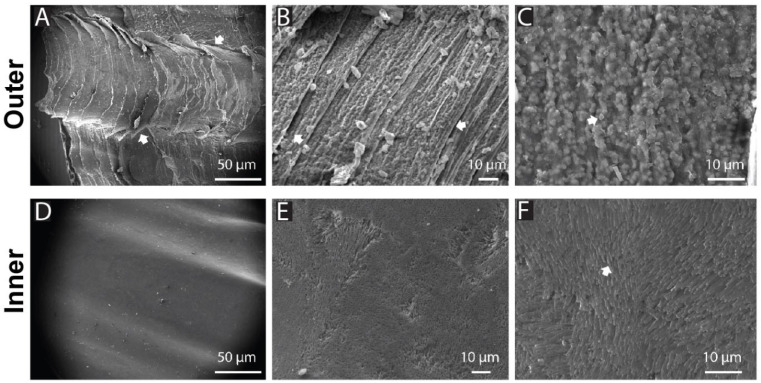
Multi-scale structure of *Argopecten purpuratus* shells, highlighting distinct morphological features on the outer and inner shell surfaces. Scanning electron microscopy (SEM) images reveal a structured, rough texture on the outer segments (**A**–**C**), while the inner segments (**D**–**F**) display a smooth surface. Arrows indicate specific regions of interest (**C**,**D**).

At various magnifications, the morphological differences between the outer and inner surfaces remained evident. SEM micrographs indicated that the outer layer exhibited a laminar configuration with notable surface roughness. Conversely, the inner layer revealed a prism-like structure, with a significantly smoother surface compared to the outer layer. At 10 µm magnification, the outer shell surface presented sheet-like and bulky structures (Figure 2B,C), while the inner face displayed a soft surface featuring foliated microstructures (Figure 2E,F). Additionally, SEM-EDS characterization of the AP shell indicated a predominant composition of calcite (47.7% O, 31.6% Ca, and 17.2% C), with 34% microporosity.

**Figure 3:**
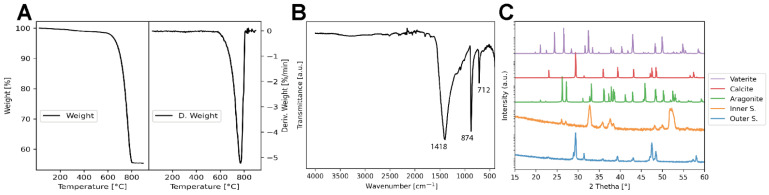
Material characterization of *Argopecten purpuratus* shells. (**A**) Thermogravimetric analysis (TGA) showing weight loss and derivative weight (%/°C) as a function of temperature. (**B**) Fourier-transform infrared (FTIR) spectra highlighting the most prominent absorption bands with corresponding wavenumbers. (**C**) X-ray diffraction (XRD) patterns of the inner (Inner S.) and outer (Outer S.) shell surfaces, compared against standard reference patterns for calcium carbonate polymorphs.

Thermogravimetric analysis showed a single major weight loss event of 43% occurring between 600 °C and 800 °C. At 900 °C, 55% of the initial sample mass remained (Figure 3A). Based on the stoichiometry of CaCO_3_ decomposition into CaO and CO_2_, the CaCO_3_ content was estimated at 98 ± 1 wt%, consistent with values reported for bivalve shells. Minor residual mass is attributed to trace inorganic impurities and the organic matrix.

FTIR analysis confirmed the presence of the calcite polymorph of CaCO_3_, displaying intense absorption bands at 874 cm^−1^, 1418 cm^−1^, and 712 cm^−1^ (Figure 3B), corresponding to the ν_2_ (out-of-plane bending), ν_3_ (asymmetric stretching), and ν_4_ (in-plane bending) vibrations of the carbonate group, respectively. Weaker bands of lower intensity were observed, attributable to minor organic residues within the shell matrix.

XRD patterns revealed clear differences between the shell surfaces (Figure 3C). The outer shell layer exhibited sharp reflections characteristic of a highly crystalline calcite structure, whereas the inner layer presented broader, less defined peaks indicative of a mixture predominantly composed of aragonite with minor amounts of vaterite and reduced crystallinity.

### 3.2. Argopecten purpuratus Shell Surface Is a Biocompatible Substrate for hGMSCs

Biocompatibility is a critical characteristic of tissue engineering scaffolds. To assess the biocompatibility of AP fragments, an MTS assay was conducted to measure metabolic activity in cells, which serves as an indicator of cell viability and adhesion.

**Figure 4:**
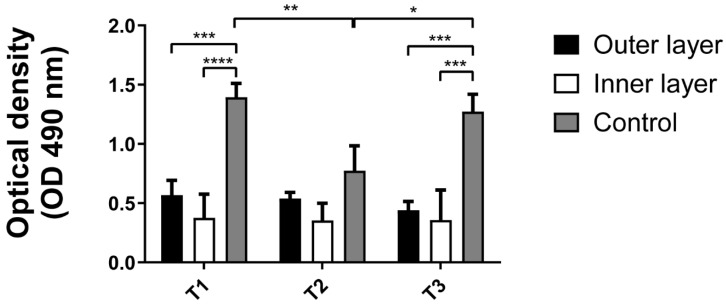
Cell viability based on adhesion in the inner and outer surfaces of *Argopecten purpuratus* (*AP*) shell fragments, demonstrating comparable values over different time points. Optical density (OD) measurements of the inner and outer shell layers were obtained using the MTS assay at culture durations of 30 min (T1), 2 h (T2), and 24 h (T3). Bars represent the mean, while whiskers indicate standard deviation (*n* = 9). Brackets denote statistical significance, with * indicating *p* < 0.05, ** for *p* < 0.01, *** for *p* < 0.001, and **** for *p* < 0.0001.

Optical density (OD) values obtained from the MTS assay are presented in Figure 4. ANOVA analysis revealed significant differences between the positive control and both layers of the AP shell (*p* < 0.001). However, no significant differences were detected between the outer and inner shell mineral surfaces at any of the tested time points. Additionally, the shell substrate demonstrated no toxicity to the cells, as they remained viable for up to 24 h.

### 3.3. Optimal Adhesion of hGMSCs on the Outer and Inner Surfaces of A. purpuratus at All Time Points Studied

**Figure 5:**
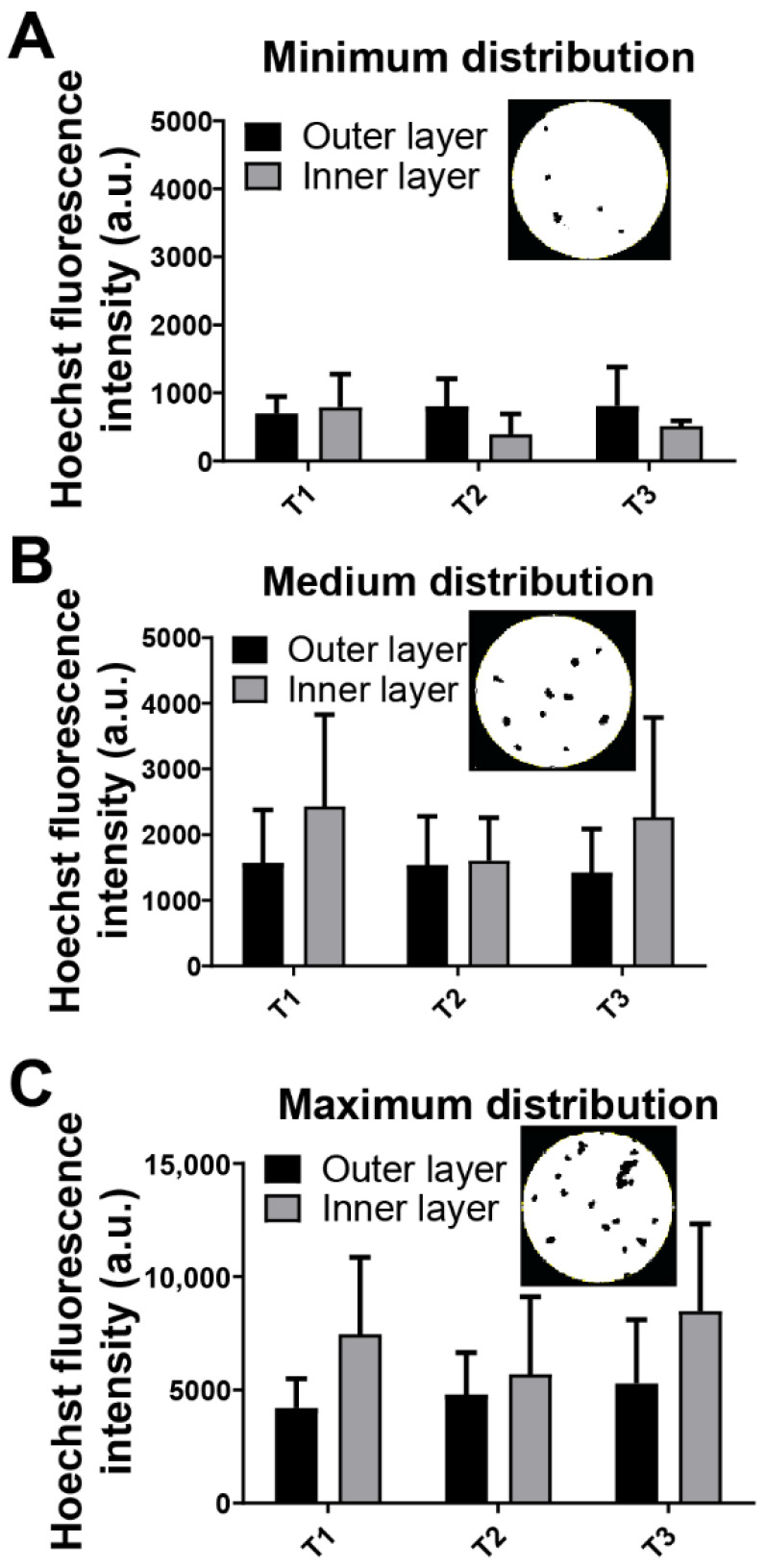
Cellular distribution of human gingival mesenchymal stem cells (hGMSC) in *Argopecten purpuratus* (*AP*) fragments at early seeding times, revealing distinct cell distribution patterns. Panels (**A**–**C**) quantify the distribution of hGMSCs on the inner and outer layers of the *AP* fragment at culture durations of 30 min (T1), 2 h (T2), and 24 h (T3). An inset image shows a representative fluorescence microscopy image of the inner layer at T1 for each distribution. Bars represent the mean, while whiskers indicate standard deviation; no significant differences were observed.

To assess the distribution of hGMSCs on selected AP shell fragments, fluorescence images using Hoechst nuclear markers (Figure 5) were analyzed with ImageJ version 1.52a. The seeding process of hGMSCs and the characteristics of the substrate influenced this cell distribution. A distribution pattern of hGMSCs was observed at early time points, categorized as minimum, medium, and maximum based on the number of cell nuclei per region of interest (ROI) analyzed. The graphs (Figure 5A–C) indicate that these distribution patterns are homogeneous across both the inner and outer shell layers at all three time points studied. Representative images of nuclear labeling with Hoechst under all conditions are presented in Figure 5. These findings align with the cell viability analyses, confirming that both the outer and inner surfaces of the AP shell effectively support hGMSC adhesion and viability.

### 3.4. The Cellular Morphology of hGMSCs Suggests Attachment to Both the Outer and Inner Surfaces of A. purpuratus During the Early Time Points

**Figure 6:**
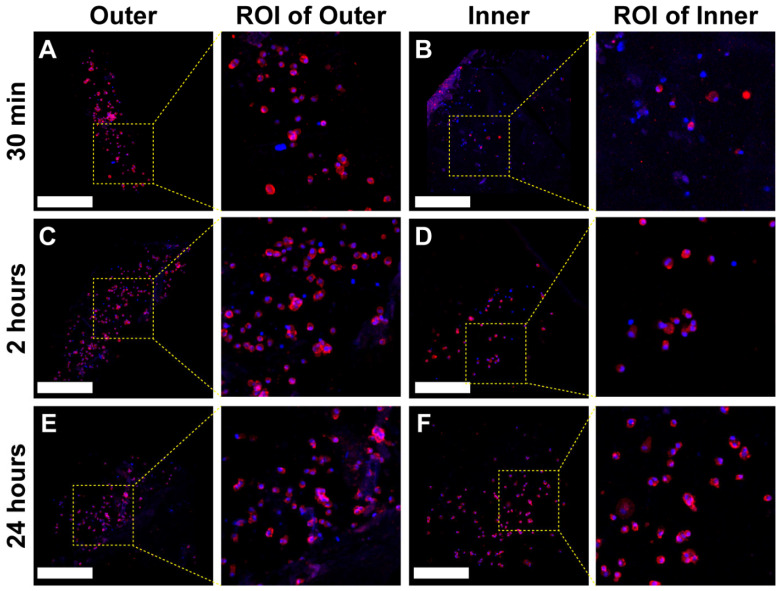
Qualitative analysis of hGMSC morphology in *Argopecten purpuratus* (*AP*) using fluorescent markers, Hoechst and Phalloidin, at early time points. A qualitative assessment of hGMSCs cultured on the inner and outer surfaces of the *AP* shell was conducted by overlaying representative confocal microscopy images labeled with Hoechst (blue) and Phalloidin (red). The images illustrate cell morphology at three time intervals: 30 min, 2 h, and 24 h, for both the outer surface (**A**,**C**,**E**) and the inner surface (**B**,**D**,**F**). Regions of interest (ROIs) are shown at higher magnification to enhance visibility of isolated cells and their structures. The scale bar (white) represents 500 µm.

Figure 6 presents the qualitative analysis of MSC morphology, featuring representative images obtained from fluorescent labeling of nuclear and actin cytoskeleton markers using Hoechst (blue) and Phalloidin (red), respectively, analyzed via confocal microscopy. Both markers exhibited specific staining patterns. Notably, in both shell surfaces at all adhesion time points, the actin cytoskeleton remained confined to the cytoplasm, displaying predominantly cortical phalloidin staining. A qualitative assessment indicated no significant differences in cell shape between the inner and outer layers across all time points studied.

**Figure 7:**
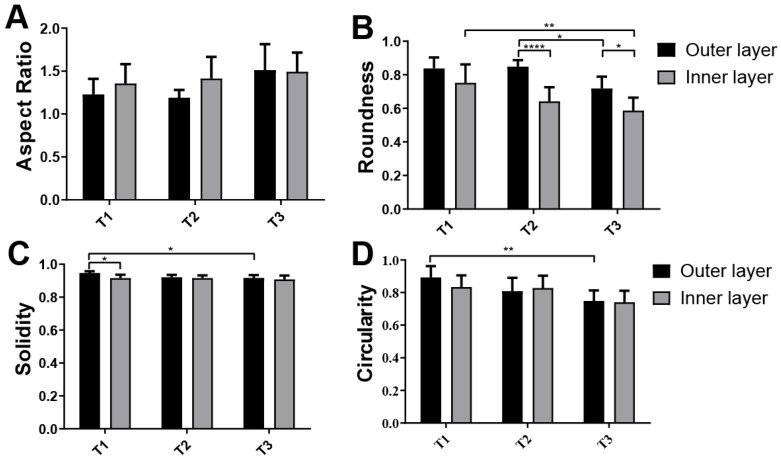
Morphological Analysis of Human Gingival Mesenchymal Stem Cells (hGMSCs) on *Argopecten purpuratus* (*AP*) Surfaces at Early Time Points Using Fluorescent Markers. This figure illustrates the quantification of hGMSC morphology on the inner and outer layers of *AP*. The analysis includes four morphological parameters: (**A**) Aspect Ratio, (**B**) Roundness, (**C**) Solidity, and (**D**) Circularity. Measurements were obtained from confocal microscopy images stained with Hoechst and Phalloidin markers. Results are presented as mean ± standard deviation (SD, N = 6) for three time points: 30 min, 2 h, and 24 h. Statistical significance was evaluated using Tukey’s test following ANOVA, with significance levels indicated by brackets (* *p* < 0.05, ** *p* < 0.01, **** *p* < 0.0001).

For quantitative analysis, cell shape was evaluated using four distinct shape indicators: aspect ratio, circularity, roundness, and solidity (Figure 7). This quantification provided a more precise assessment of cellular morphological changes. Furthermore, three of the four factors analyzed (roundness, solidity, and circularity) showed significant changes within 24 h (*p* < 0.05), suggesting that by this time, the cells began transitioning from a rounded morphology to a more flattened shape (Figure 7B–D).

**Figure 8:**
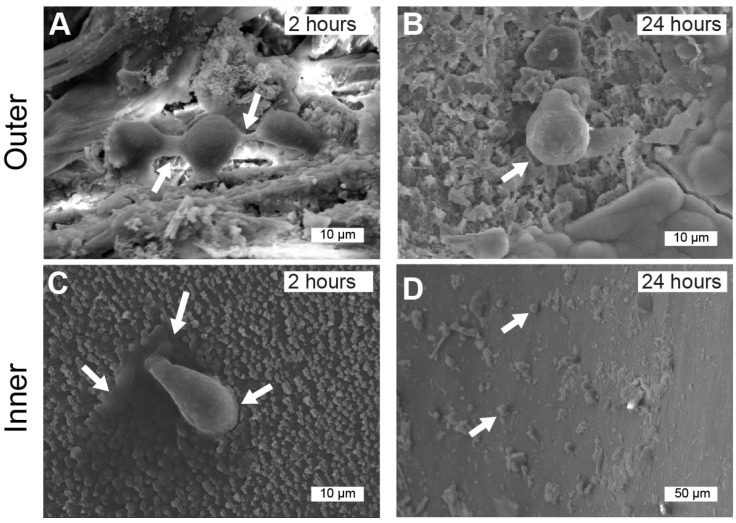
Morphological Analysis of Human Gingival Mesenchymal Stem Cells (hGMSCs) on the Inner and Outer Layers of *Argopecten purpuratus* (*AP*) Using Scanning Electron Microscopy (SEM). This figure presents representative SEM images of *AP* fragments from both outer and inner surfaces, captured at two time points: 2 h and 24 h. Higher magnification images (**A**–**C**) illustrate hGMSCs with a rounded morphology (indicated by arrows) adhering to the surface of the fragments. Image (**A**) highlights interactions between cells through cytoplasmic extensions, suggesting cell-to-cell contact. (**B**) Cell with circular morphology on the substrate (arrows). Additionally, image (**C**) shows the adhesion of hGMSCs characterized by circumferential lamellipodia (dark gray areas with irregular edges surrounding the cell, indicated by arrows) after 2 h of culture. (**D**) Low magnification image of the adhesion at 24 h showing cells (hGMSC) with morphology (arrowheads).

A further analysis of cell adhesion and morphology of hGMSCs was conducted using scanning electron microscopy (SEM). Shell granules obtained from *A. purpuratus*, seeded with cells, were examined for cell adhesion after 2 h and 24 h in culture. Cells were observed to attach to the seashell biomaterial, as illustrated in Figure 8, indicating optimal adhesion to the substrate, which is a crucial precursor to cell proliferation and differentiation. SEM images revealed that cells were connected on the outer surface after 2 h and on the inner surface after 24 h (Figure 8A and Figure 8D, respectively). The analysis demonstrated that hGMSCs were spreading and forming connections with other cells, with projections from their cytoplasmic membranes suggesting attachment to the mineral surface.

At the 2-h mark, cells on the outer shell surface maintained a rounded morphology, adhering to the substrate through a small region of the cell membrane while also exhibiting some cell–cell interactions (indicated by white arrows). Additionally, some cells displayed protrusion-like structures. In contrast, cells attached to the inner shell surface exhibited a water-drop morphology, displaying lamellipodia structures (white arrows in Figure 8C), while others remained rounded at the same time point.

### 3.5. Cellular Morphology of hGMSCs Demonstrates Attachment to Both the Outer and Inner Surfaces of A. purpuratus at Later Time Points

**Figure 9:**
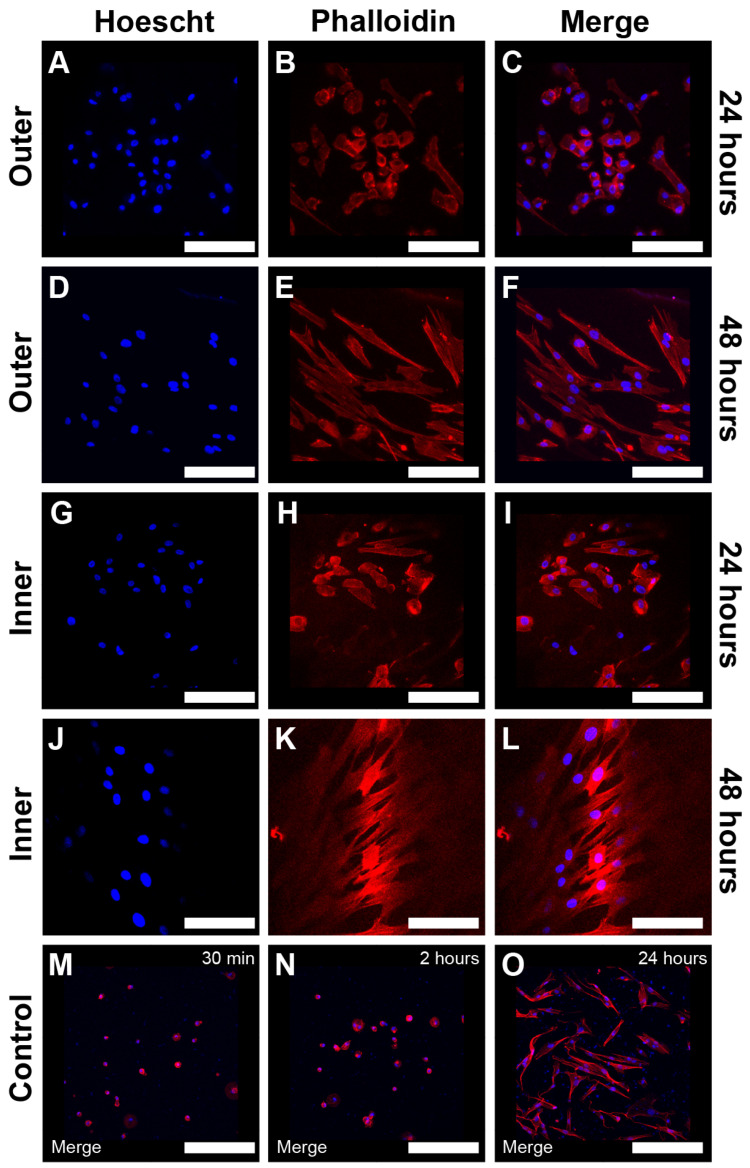
Adhesion of Human Gingival Mesenchymal Stem Cells (hGMSCs) on Inner and Outer Surfaces of *Argopecten purpuratus* (*AP*) After 48 h in Culture, as Assessed by Fluorescent Markers. This panel presents a qualitative analysis of hGMSC morphology on the inner and outer surfaces of *AP*, using confocal microscopy with Hoechst (blue) and Phalloidin (red) fluorescent markers. Representative images were superimposed (Merge) to illustrate cellular characteristics at two time points: 24 h and 48 h. Images (**A**–**C**,**G**–**I**) depict cells at 24 h displaying a more compact morphology, while images (**D**–**F**,**J**–**L**) show a more elongated structure at 48 h. Control images include the superimposed (Merge) representations of hGMSCs in culture at 30 min (**M**), 2 h (**N**), and 24 h (**O**). The scale bars (white) indicate 500 µm.

Morphological changes and qualitative cell counts were analyzed at longer post-seeding intervals. hGMSCs stained with fluorescent markers for nuclei and actin cytoskeleton were evaluated at 24 h, 48 h, and 4 weeks post-seeding. At 24 h, both shell surfaces exhibited a mix of rounded cells and cells with fibroblast-like spindle shapes (Figure 9A–C,G–I). In contrast, after 48 h, all cells displayed a fully fibroblast-like morphology, indicating complete cytoplasmic spreading across the mineral surfaces (Figure 9D–F,J–L).

**Figure 10:**
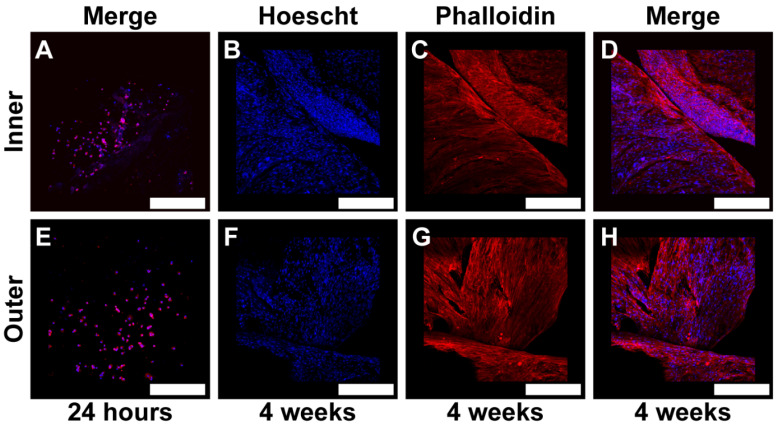
Adhesion of Human Gingival Mesenchymal Stem Cells (hGMSCs) on *Argopecten purpuratus* (*AP*) at Early Time Points and After Four Weeks, Assessed Using Fluorescent Markers. This figure presents a qualitative analysis of hGMSC adhesion on the inner and outer layers of *AP*, utilizing confocal microscopy with Hoechst (blue) and Phalloidin (red) fluorescent markers. Representative images were superimposed (Merge) to evaluate cell adhesion at 24 h (**A**,**E**) and after four weeks (**B**–**D**,**F**–**H**). Observations include the inner surface in panels (**A**–**D**) and the outer surface in panels (**E**–**H**). The scale bars (white) indicate 500 µm.

After 4 weeks of attachment, a complete covering of both the outer and inner shell surfaces by hGMSCs was observed (Figure 10B–D,F–H). These findings suggest that the seashell surface not only provides a suitable substrate for cell adhesion but also supports cell proliferation, as evidenced by the increased fluorescence intensity of nuclear and actin cytoskeleton staining.

## 4. Discussion

The increasing interest in marine-derived biomaterials stems from their unique chemical, mechanical, and topographic properties, which render them highly suitable for biomedical applications, particularly in bone tissue engineering. Recent research has highlighted the potential of seashells from various marine organisms as sustainable and cost-effective sources of high-quality biomaterials [22]. This study adds to this growing body of knowledge by investigating the biocompatibility and viability of using the *Argopecten purpuratus* shells as a biomaterial to support the adhesion, viability, and proliferation of human gingival mesenchymal stem cells (hGMSCs).

*Agropecten purpuratus* (*AP*) shells are primarily composed of calcitic calcium carbonate, with approximately 96.5% composition, aligning with previous studies on bivalve species [23,24]. Structural analysis indicates that the *A. purpuratus* shell possesses densely packed microstructures, featuring an outer surface marked by irregular topographical elements such as grooves and striae, while the inner surface exhibits a smoother, foliated-like structure. These varied surface characteristics provide different topographical cues that can significantly influence cell adhesion, a crucial aspect of tissue engineering.

The chemical composition and crystalline structure identified in *AP* shells are comparable to findings in other bivalve species. FTIR spectra dominated by calcite-associated carbonate bands and minor signals from organic residues are typical of mollusk shells, which contain <5% organic matrix embedded in a predominantly CaCO_3_ mineral phase [25,26]. The presence of weaker bands likely reflects residual proteins and polysaccharides known to participate in biomineralization [27]. XRD results further highlight the hierarchical mineral organization of *AP* shells, with the outer layer composed of highly crystalline calcite and the inner layer containing mixed polymorphs (vaterite and aragonite). The high CaCO_3_ content (≈98 wt%) observed from TGA is consistent with reports for other marine-derived shells, such as Anadara granosa and Strombus canarium, which typically exceed 95 wt% CaCO_3_ [24,27]. This compositional purity underscores the potential of *AP* shells as a sustainable CaCO_3_ source for biomedical applications.

Human gingival mesenchymal stem cells (hGMSCs) can adhere to both the inner and outer surfaces of the *AP* shell without significant differences in cell viability. After 24 h of culture, the cells were uniformly distributed across the mineral surfaces and exhibited similar morphologies, indicating that the scallops shell surface is biocompatible and capable of supporting cell adhesion. This finding is crucial, as biocompatibility is a fundamental requirement for any biomaterial intended for in vivo applications, where appropriate cellular responses are essential [28,29], as revealed by the dynamic morphological indicators analysis of our results. Moreover, Figure 5 shows that at different time the minimal, medium and maxima distribution is not statistically significant for both inner and outer surfaces, showing the similarity of the performance in both surfaces.

Despite both surfaces facilitating cell adhesion, scanning electron microscopy (SEM) analysis revealed differences in the morphology of hGMSCs on the distinct shell surfaces. On the outer shell surface, the cells displayed a rounded morphology with active membrane interactions at early time points. In contrast, the inner surface presented cells with a more planar morphology and limited membrane contact at later time points. These observations suggest that the microstructure of the shell surfaces significantly influences cell adhesion, aligning with the idea that topographical features can modulate protein adsorption and the dynamics of cell adhesion [16].

Extensive cytoplasmic spreading across the mineral surface was observed at 48 h of culture. Morphological changes occurred between 24 and 48 h, highlighting a transition from a more circular cell shape to an elongated form, which emphasizes the effective support provided by the mineral surface for cell adhesion. Mollusks are soft-bodied metazoans characterized by outer calcified shields that display a diverse range of biomineral structures and morphologies. The formation of mollusk shells appears to be a biologically controlled mineralization process facilitated by an extracellular organic matrix. This matrix plays a critical role in the nucleation, orientation, and morphology of the resulting crystals, producing structures that differ significantly from chemically synthesized calcium carbonate crystals [30,31]. Moreover, the organic matrix of the shell provides a three-dimensional framework that guides crystal growth in specific directions and regulates their growth as needed, resulting in a variety of biomineral microstructures [31]. The diversity of mollusk shell microstructures seems to be constrained by mineral composition; for instance, typical calcite structures are fibrous, prismatic, and foliated [31], and the microstructures observed in both the inner and outer surfaces of the scallop shells fall within these categories. However, further investigation is needed to determine how the microstructures of seashell biominerals affect their ability to support mesenchymal stem cell (MSC) adhesion, proliferation, and differentiation.

One of the most significant findings of this study is that the *AP* shell not only supports cell adhesion but also promotes long-term cell proliferation. After four weeks of culture, hGMSCs had completely covered both the inner and outer shell surfaces, indicating robust cell proliferation (Figure 10). This prolonged viability and proliferation further underscore the potential of the *AP* shells as a viable bio-scaffold for tissue engineering. Future experiments should address a comparison between the studied natural source and commercial bioceramic based biomaterials (e.g., HA, β-TCP). Additionally, the ability of the shell to maintain cell viability over extended periods suggests that it may also support other cellular processes, such as differentiation, although this was not specifically addressed in this study (see Figure 1, Supplementary Material). Thus, our results agree with previous research showing that marine-derived calcium carbonate materials possess excellent osteoconductivity, biocompatibility, and osteogenic potential, making them promising candidates for bone tissue regeneration [4,32,33].

Marine-derived calcium carbonate biomaterials, such as *AP* shells, are gaining attention due to their biocompatibility, structural similarity to mineralized tissues, and potential for chemical transformation into hydroxyapatite while preserving hierarchical micro- and nanoscale architecture. Previous studies have demonstrated that natural CaCO_3_ from seashells can serve as a precursor for hydroxyapatite synthesis or as a functional filler in composite scaffolds, improving mechanical performance and biological integration in bone tissue engineering. *AP* shells are also a sustainable and abundant by-product of aquaculture along the Humboldt Current, where scallop farming generates large quantities of shell waste [34]. Reusing this material as a biomedical substrate provides an environmentally sustainable approach consistent with circular economy principles [35,36,37,38]. While the present study focused on cytocompatibility and cell-supportive properties, further research is necessary to evaluate functional differentiation outcomes and explore the full potential of *AP* shells in clinical regenerative applications [28].

Moreover, several research groups have successfully improved the material properties of seashell-derived biomaterials through chemical transformations while maintaining the intrinsic morphological structure of the original shells. Natural calcium carbonate (CaCO_3_) sourced from seashells has been shown to be an effective precursor for producing hydroxyapatite (HA), a process well-documented in the literature [39,40]. This transformation enables the material to retain its biocompatibility and osteoconductivity, making it highly applicable in bone tissue engineering. Additionally, natural CaCO_3_ has been effectively utilized as an active filler in 3D scaffold architectures, enhancing both the mechanical and biological properties of the scaffolds [41]. This strategy capitalizes on the inherent advantages of natural materials while optimizing their properties for advanced biomedical applications.

The mechanical properties of a biomaterial substrate can play a critical role in influencing cell behavior, including adhesion, morphology, and differentiation. While the present study focused on cytocompatibility and cell–material interactions, including detailed analysis of cell morphology on *AP* shell surfaces, we did not perform full mechanical characterization. In ongoing work, the local mechanical response of *AP* shells is being evaluated using microindentation and hierarchical structural analysis, which will provide complementary insights into how substrate stiffness interacts with micro- and nanoscale surface features to influence cellular behavior. This future work will help clarify the relationship between mechanical properties and stem cell responses on marine-derived biomaterials.

The present study demonstrates that *AP* shells provide a cytocompatible substrate capable of supporting hGMSC adhesion, viability, and long-term proliferation, with cell morphology adapting to surface micro- and nanoscale features. These results confirm that *AP* shells are suitable for applications requiring stable cell–material interactions. It should be noted, however, that osteogenic and chondrogenic differentiation were not evaluated in this work, and therefore the conclusions are limited to biocompatibility and cell-supportive properties. Future studies should investigate lineage-specific differentiation by assessing early and late markers, including ALP activity, RUNX2, osteocalcin, and mineralized matrix deposition for osteogenesis, and SOX9 and collagen type II for chondrogenesis. Previous studies suggest that marine CaCO_3_ templates can enhance osteogenesis via Ca^2+^ release from bioactive organic matrix components, and that CaCO_3_ nanoparticles can act as carriers for osteogenic factors such as BMP-2, providing sustained release that promotes osteogenic signaling and *ALP* activity. These findings provide a framework for future work to evaluate the functional differentiation potential of *AP* shells while maintaining the clear evidence of cytocompatibility established in this study.

## 5. Conclusions

This study highlights the potential of *Argopecten purpuratus* (*AP*) scallop shells as a biocompatible scaffold for human gingival mesenchymal stem cells (hGMSCs), demonstrating their capacity to support cell adhesion and long-term proliferation. The unique microstructural features of the *AP* shell, primarily composed of calcium carbonate, significantly influence cell behavior, underscoring the importance of topographical cues in tissue engineering applications. The ability of hGMSCs to proliferate extensively over a four-week period further suggests that *AP* shells could serve as effective bio-scaffolds for regenerative therapies. Additionally, the utilization of marine-derived biomaterials aligns with sustainable practices, addressing waste management in aquaculture while providing a cost-effective solution for biomedical applications. Future investigations should focus on the osteogenic potential of these shells, as well as their integration with other biomaterials to enhance their efficacy in bone regeneration strategies. This research contributes to the growing field of marine biomaterials, emphasizing their viability in advancing eco-friendly and innovative approaches to tissue engineering.

## Data Availability

The original contributions presented in the study are included in the article, further inquiries can be directed to the corresponding author.

## Supplementary Materials

The following supporting information can be downloaded at: https://www.mdpi.com/article/10.3390/jfb17040164/s1. The files represent Figure 9 and Figure 10 of the unmerged evaluation.
