# Supplementary figures and images for: Characterization of Argopecten purpuratus Shells as Marine-Derived Bioceramics: Microstructural and Biological Insights for Tissue Engineering Applications
Source: J Funct Biomater. 2026 Apr 1;17(4):164. doi: 10.3390/jfb17040164 (PMC13117402; doi:10.3390/jfb17040164)

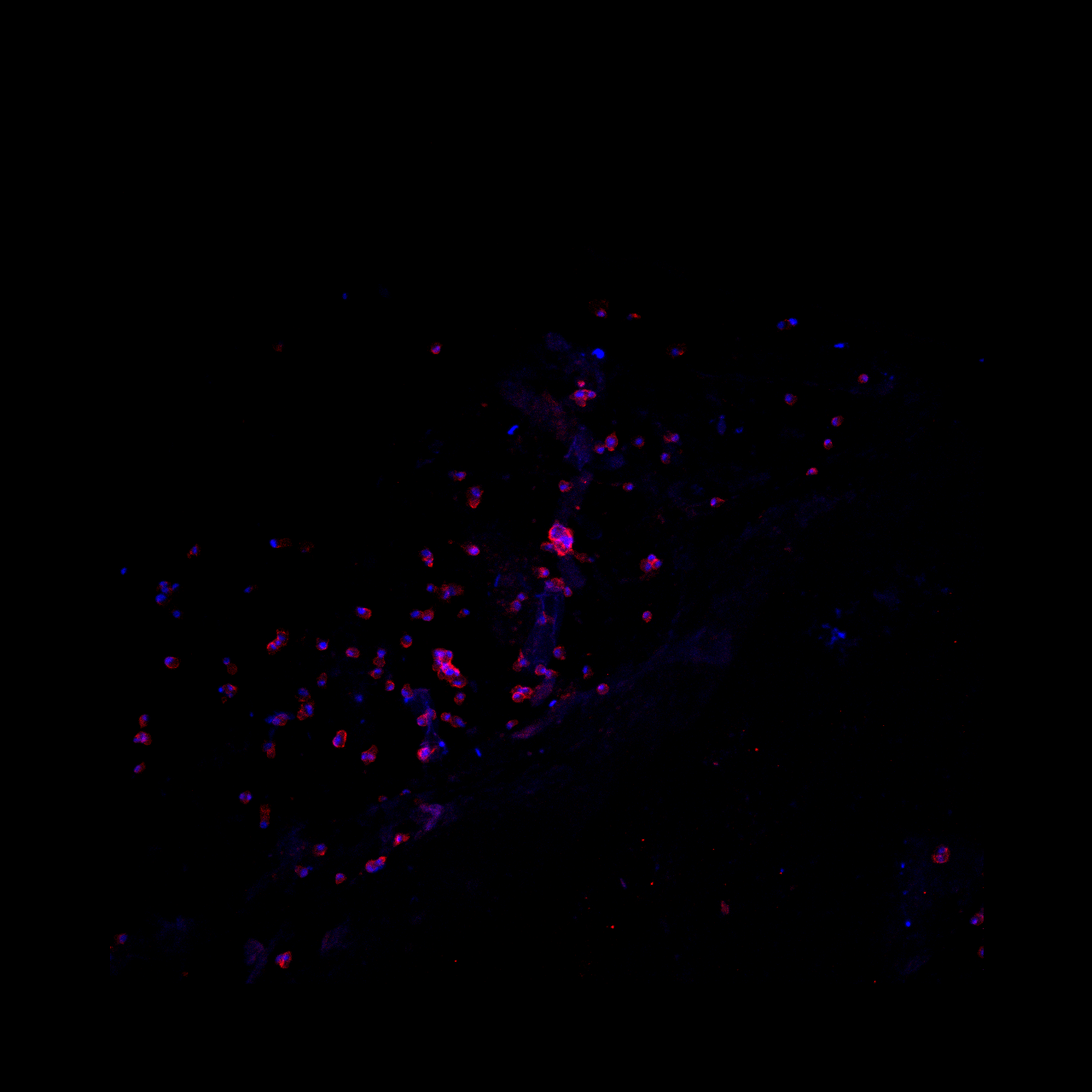

Supplement: Supplementary file 1 [file jfb-17-00164-s001.zip › Figure 10A.tif]

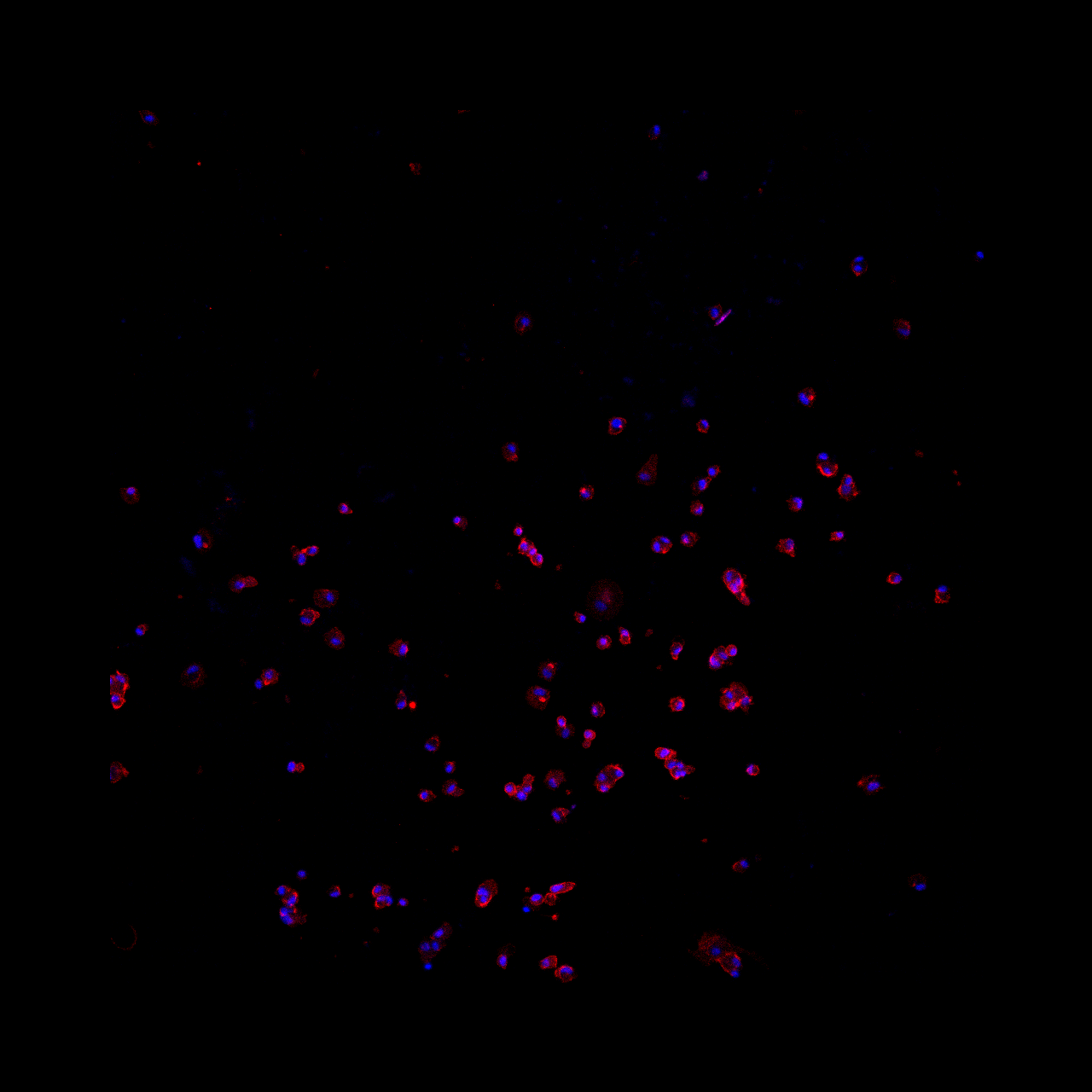

Supplement: Supplementary file 1 [file jfb-17-00164-s001.zip › Figure 10E.tif]

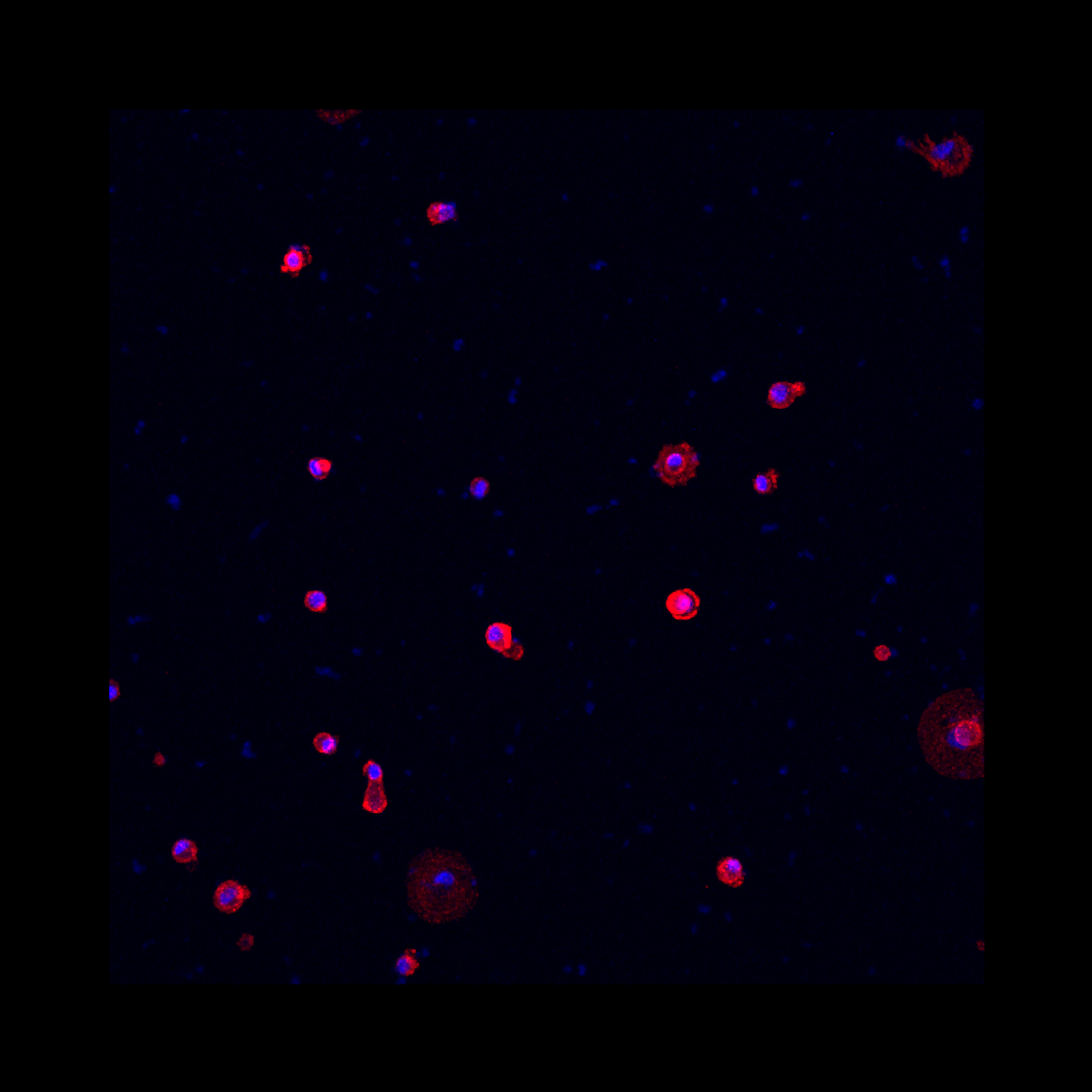

Supplement: Supplementary file 1 [file jfb-17-00164-s001.zip › Figure 9M.tif]

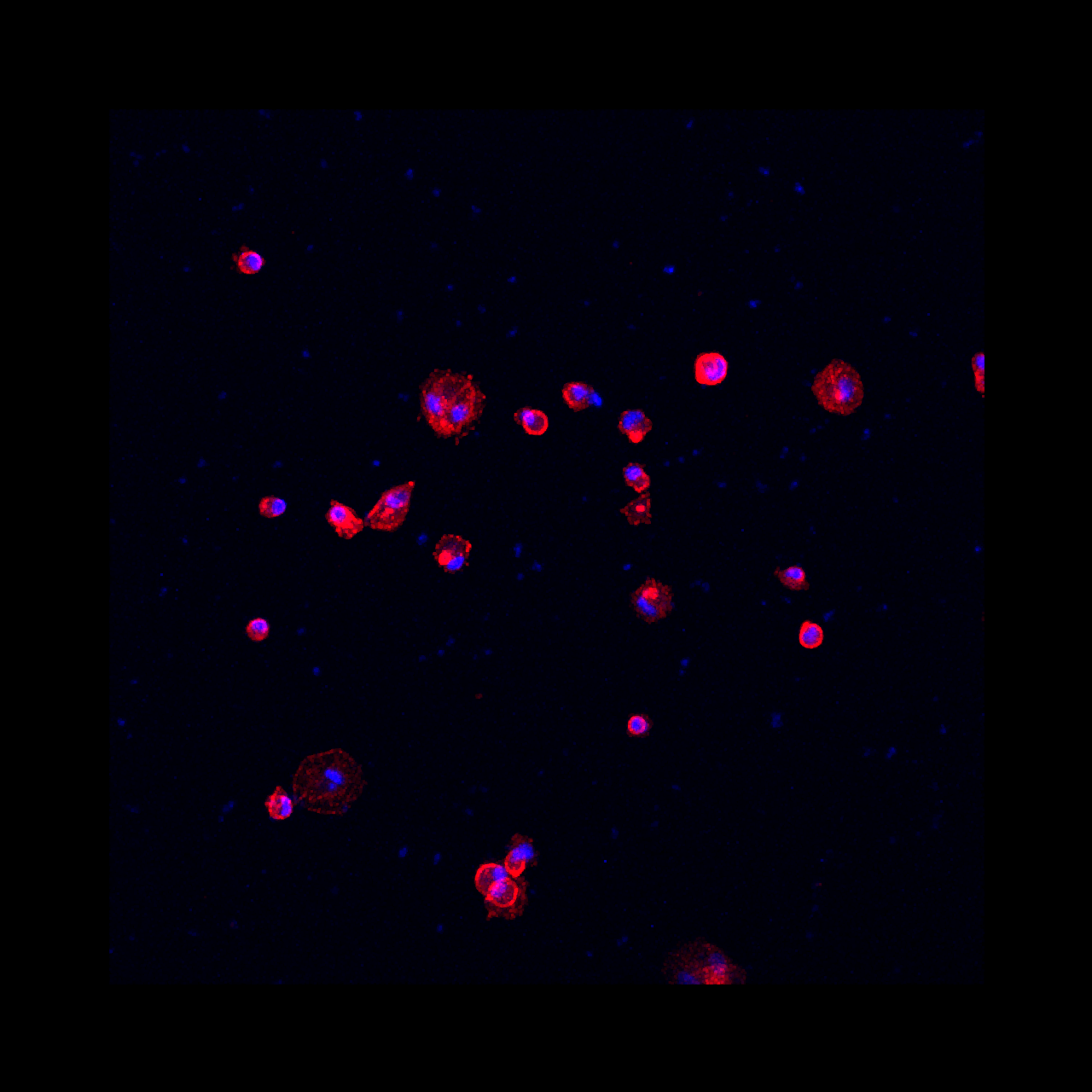

Supplement: Supplementary file 1 [file jfb-17-00164-s001.zip › Figure 9N.tif]

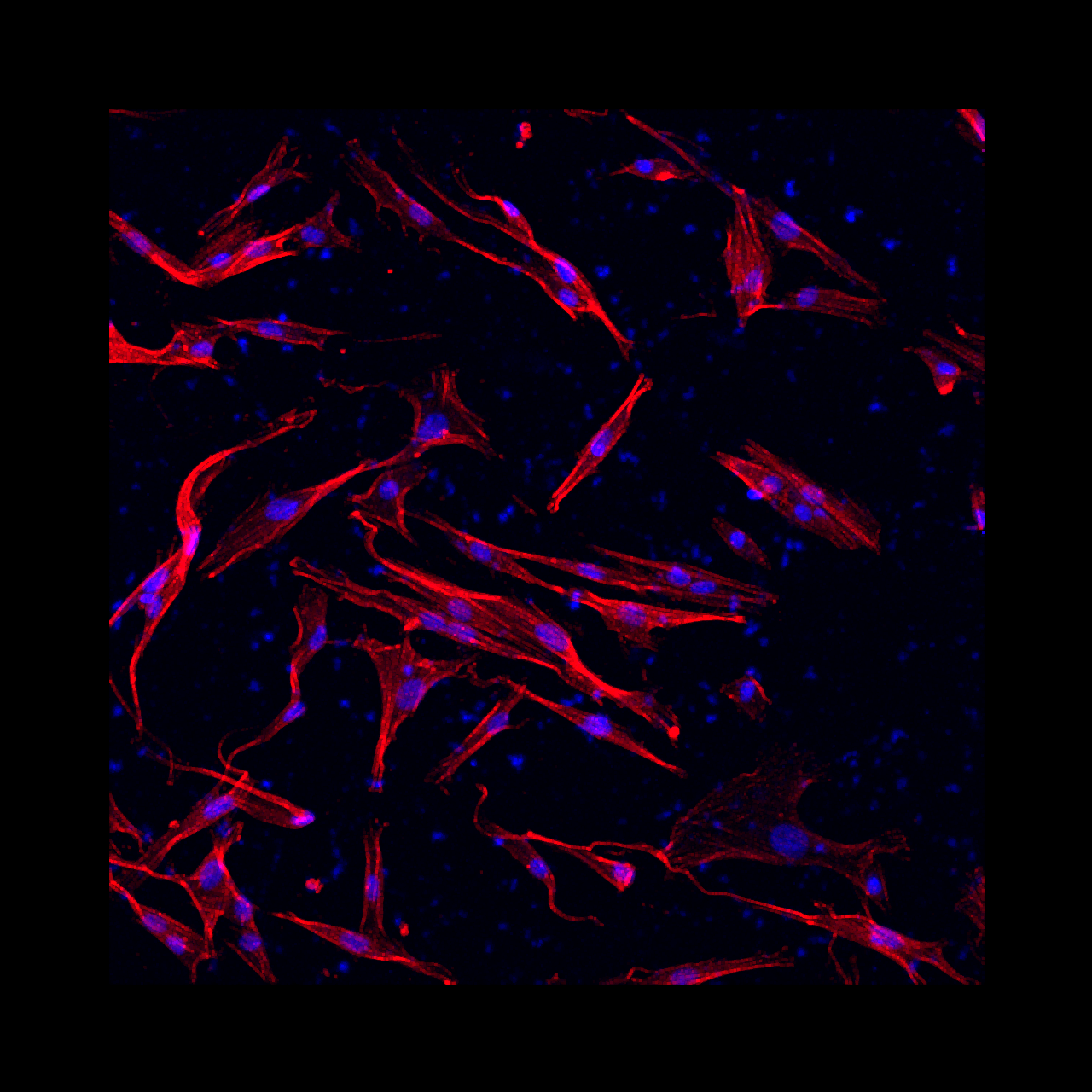

Supplement: Supplementary file 1 [file jfb-17-00164-s001.zip › Figure 9O.tif]
